# Prevalence of the Metabolic Syndrome among rural original adults in NingXia, China

**DOI:** 10.1186/1471-2458-10-140

**Published:** 2010-03-17

**Authors:** Zhao Yi, Jin Jing, Liu Xiu-ying, Xu Hongxia, Yang Jianjun, Zhang Yuhong

**Affiliations:** 1 Public Health School, Ningxia Medical University 1160 Shengli Street, xingqing district, 750004 Yinchuan, Ningxia, PR China

## Abstract

**Background:**

Metabolic syndrome (MS) is combination of medical disorders that increase people's risk for cardiovascular disease and diabetes mellitus. Little data exists on the prevalence of MS of rural original adults in Ningxia of China.

**Methods:**

A cross-sectional survey method was used and the participants were interviewed by trained health workers under a structured questionnaire in rural of Ningxia in 2008. The number of research subjects was 1612. MS was defined by International Diabetes Federation IDF (2005).

**Results:**

The age-adjusted prevalence of the metabolic syndrome was 11.8%, whereas ethnic-specific prevalence was 10.3% in Han ethnic group and 13.7% in Hui ethnic group. Components of MS and MS were more common in Hui ethnic group than Han ethnic group. The mean levels and prevalence of abnormal value increased with increasing age in both ethnic groups (Cochran-Artimage test for trend, Hui ethnic group P < 0.05, Han ethnic group P < 0.01).

**Conclusions:**

The prevalence of MS was high in rural residents' adults in Ningxia. Clustering of MS components and MS was increased with age. The components of MS have big differences among different ethnic groups.

## Background

It is well accepted that the metabolic syndrome is a condition that promotes atherosclerosis and increases the risk of cardiovascular disease and diabetes mellitus [1-3]. Each abnormality of syndrome (ie, atherogenic dyslipidemia, a prothrombotic state, insulin resistance, hypertension, and abdominal obesity) can enhances the risk of atherosclerosis independently, but when clustered together, these metabolic disorders are increasingly atherogenic and enhance the risk of cardiovascular morbidity and mortality [2].

Several studies had illustrated a high prevalence of diabetes, impaired glucose tolerance, obesity, and hypertension among Chinese [4-9]. However, all of the studies focused on estimating the population distribution of the all ethnics and major risk factors for CVDs. There was a lack of studies available to provide information on the prevalence of MS in rural population in China, especially in the relatively un-developed area likes Ningxia. In addition to the development of area, the lifestyle closely linked with the specific ethnic group traditions could be another important factor to influence the prevalence of MS. Finally, we supposed that such kind of specific participants, with average but stable income and living condition, lower educational background and lower health awareness in rural area in Ningxia of China, could provide us opportunity to get more confident results. Here we used the working definition provided by the International diabetes federation (IDF2005) to estimate the prevalence of the metabolic syndrome between Han ethnic group and Hui ethnic group among Ningxia rural original peoples.

## Methods

### Study population

The study was a chronic disease and nutrition survey carried out in the province of Ningxia in china (100% rural area). Data collection started in May 2008 and ended in March 2009. To control the precision of sampling, stratified cluster sampling was applied. First, different levels of economic income region (Wuzhong city and Guyuan city) were selected among the total five regions in Ningxia, then one Hui ethnic village and one Han ethnic village were randomly selected in each region. Sampling database was based on the age-ethnic distribution of the Ningxia province by local government Statistical department (census 2005). The target group was all Ningxia original rural peoples aged ≥ 25 years who resided in countryside for at least fourth generations before the date of survey. During the study period, 2082 inhabitants from the area and without any clinical evidence for cardiovascular disease were randomly selected by computer to participate in the study; finally 1612 were enrolled through whole study. Thus, 22.5% of those who were eligible did not participate due to several reasons (eg, lack of time or unexpected reasons that forced them to cancel the interview) that were not related to the hypothesis tested. Among them, 928 were Han ethnic group and 684 were Hui ethnic group. The subjects did not report chronic viral infection, cold or flu, acute respiratory infection, dental problems, or any type of surgery in the week preceding the study. All participants were interviewed by trained person with a standard closed-ended questionnaire.

The study protocol has been approved by The Medical Ethics Review Committee of Ningxia Medical University and there are no activities against ethical policies in China. All participants have signed the consent on enrolment after they received written and verbal information about the trial.

### Anthropometric, clinical, and biochemistry characterizations

Standing height was measured once using a portable ruler (made in china), body weight was also measured once using a weight scale (made in china). All measurements were performed by the study investigators. Body mass index (BMI) was calculated as kg/m^2^. Obesity was defined as BMI ≥ 30 according to WHO standard guidelines [10]. Arterial blood pressure was measured three times in sitting position. All participants were at rest at least 30 minutes before the measurement. Patients with average blood pressure ≥ 140/90 mmHg or taking antihypertensive medication were classified as hypertensive. Blood samples were collected from the antecubital vein between 6 and 8 Am, after 10 hours of fasting and avoidance of alcohol. Two sets of fasting blood samples were collected from each subject in sodium fluoride potassium oxalate tubes (for glucose) and lithium heparin vacuum tubes (for lipids). Samples were centrifuged at the survey site, and plasma was transferred to separate tubes and labelled and transferred immediately in cold boxes filled with ice (2-8°C). Finally samples were frozen at -20°C for further analysis afterwards. HDL cholesterol and triglyceride levels were measured using chromatographic enzyme method (Automatic biochemical analyzer OLYMPUS AU400). The intra and inter-assay coefficient of variation of cholesterol and triglycerides did not exceed 4%. Hypercholesterolemia was defined as total serum cholesterol levels ≥ 5.72 mmol/L. Blood glucose level were measured immediately after on-site collection with Blood Glucose Meter (Life Scan inc. Milpitas, CA 95035 U.S.A.)

### The Metabolic Syndromes

In International Diabetes Federation (IDF 2005) definition, a participant was defined as having metabolic syndrome if he or she had central obesity (waist circumference ≥ 90 cm for men or ≥ 80 cm for women in Chinese people) plus at least 2 of the following criteria: (1) triglyceride level ≥ 1.7 mmol/L; (2) reduced HDL-C levels of less than 1.03 mmol/L in men, less than 1.29 mmol/l in women; (3) raised systolic or diastolic blood pressure of 130/85 mm Hg or higher or previously diagnosed hypertension; and (4) raised fasting plasma glucose level of 5.6 mmol/L or higher or previously diagnosed type 2 diabetes mellitus.

### Statistical analysis

Continuous variables are presented as mean values ± standard deviation and categorical variables are presented as absolute and relative frequencies or prevalence. Associations between categorical variables were tested by χ^2 ^test and Cochran-Artimage test for trend analysis, while differences in biochemical and clinical indexes between categorical variables were tested by ANOVA. All reported *p *values are based on two-sided tests with a significance level of 5%. All data were input into Epidata first and then exported into SPSS. SPSS (version 14.0 SPSS Corp, College Station, NX) was used for all statistical analysis. To facilitate comparisons with other published data, all prevalence rates were age-adjusted by direct method [11] with step size of 10-years using the Word Stand Population [12].

## Results

**Figure 1:**
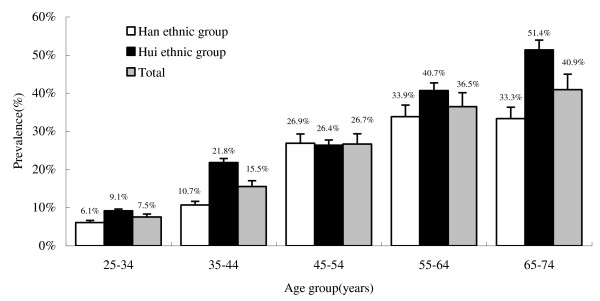
**Age-specific prevalence of the metabolic syndrome among 1612 rural adults at least 25 years**. Point estimates and 95% confidence intervals are shown.

Table 1 includes the basic social demographic information and also the key clinical characterization results for the participants in this study. As shown in Table 2, different MS components and MS values were presented in the categories of Han ethnic, Hui ethnic and total. The age-adjusted prevalence of the MS was 11.8%, whereas ethnic-specific prevalence was 10.3% in Han ethnic group and 13.7% in Hui ethnic group base on WASR1985. Ethnic-specific crude prevalence of MS were 20.0% and 24.7% in Han ethnic group and Hui ethnic group, respectively (p = 0.022). A trend curve of the prevalence of the MS was observed by age groups in all participants (Figure 1). In Han ethnic group: 6.1% in those < 35 years old, 10.7% in those 35 to 44 years old, 26.9% in those 45 to 54 years old, 33.3% in those 55 to 64 years old, and 33.9% in those over 64 years old; in Hui ethnic group: 9.1% in those < 35 years old, 21.8% in those 35 to 44 years old, 26.4% in those 45 to 54 years old, 40.7% in those 55 to 64 years old, and 51.4% in those over 64 years old. The age-adjusted prevalence of obesity was markedly higher among Hui ethnic group than Han ethnic group (2.2% vs 1.3%) (see Table 2). All other components of MS were also more common in Hui ethnic group. Of all components of the MS, low HDL cholesterol was the most common abnormality in the studied population, 27.7% of individuals had low HDL cholesterol, approximately 1.6% of the populations had obesity, 24.2% of the population had high FPG and 8.5% of the population had hyper-triglycerides. The age-specific mean values and proportions of subjects with abnormality in components of the MS, as well as BMI, are given in additional file 1. The mean values and prevalence of MS components increased with increasing age in both ethnic groups (Cochran-Artimage test for trend, Hui ethnic group *P *< 0.05, Han ethnic group *P *< 0.01).

**Table 1:** Socio-demographics, clinical characteristic, lipid profile and fasting glucose of study participants

	Han ethnic group (n = 928)	Hui ethnic group (n = 684)	*P *value
**Demographic and lifestyle variables**	**Absolute and Relative (%) Frequency**		
Age group			
25-	181(19.5%)	164(24.0%)	χ^2 ^= 7.128 *P *= 0.129
35-	262(28.2%)	202(29.5%)	
45-	242(26.1%)	163(23.8%)	
55-	192(20.7%)	118(17.3%)	
65-80	51(5.5%)	37(5.4%)	
**Clinical characteristics**	**Mean ± SD**		
	
Waist (cm)	78.6 ± 8.9	79.7 ± 9.8	**0.026**
BMI (Kg/m^2^)	23.1 ± 3.1	23.7 ± 3.3	**< 0.001**
Diastolic blood pressure (mmHg)	78.7 ± 11.4	80.4 ± 20.0	**0.038**
Systolic blood pressure (mmHg)	122.6 ± 20.3	122.5 ± 24.7	0.926
Total serum cholesterol (mmol/L)	3.8 ± 0.8	3.9 ± 0.8	0.299
High-density lipoprotein cholesterol (mmol/L)	1.2 ± 0.3	1.1 ± 0.2	**< 0.001**
Triglyceride (mmol/L)	1.2 ± 0.7	1.2 ± 0.8	0.172
Blood glucose (mmol/L)	5.6 ± 1.2	5.6 ± 1.1	0.936

**SD **= standard deviation, See RESEARCH DESIGN AND METHODS for description of criteria of the metabolic syndrome.

**Table 2:** Age-adjusted prevalence of individual metabolic abnormalities of the metabolic syndrome among rural adults in Ningxia aged ≥ 25.

	Absolute and Prevalence (%, 95%CI)		
	
	Han ethnic group	Hui ethnic group	Total
n	928	684	1612

Overweight	234 (14.7, 12.4-16.9)	224 (17.9, 15.3-20.7)	458 (15.6, 13.8-17.4)
Obesity	23 (1.3, 0.6-2.0)	29 (2.2, 1.1-3.3)	52 (1.6, 1.0 -2.2)
Low HDL cholesterol	429 (25.7,22.9-28.5)	376 (30.4, 26.9-33.8)	805 (27.7, 25.5-29.9)
Hypertension	364 (21.1, 18.5-23.7)	257 (21.2, 18.1-24.2)	621 (21.1, 19.2-23.2)
Hyper-triglyceridemia	129 (7.6, 5.9-9.3)	117 (9.7, 7.5-11.9)	246 (8.5, 7.1-9.8)
High FPG or on medication	405 (23.7,20.9-26.4)	308 (24.9,21.7-28.2)	713 (24.2, 22.1-26.3)
Metabolic syndrome (IDF)	186 (10.3, 8.4-12.3)	169 (13.7, 11.2-16.3)	355 (11.8, 10.2-13.4)

Over weight: BMI≥ 25 and BMI < 30; Obesity: BMI≥ 30; Low HDL cholesterol: HDL-C < 1.03 mmol/L in men, HDL-C < 1.29 mmol/l in women; Hypertension: systolic or diastolic blood pressure of 130/85 mm Hg or higher or previously diagnosed hypertension; Hyper-triglyceridemia: defined as total serum cholesterol levels 5.72 mmol/L; High FPG or on medication: fasting plasma glucose ≥ 5.6 mmol/L or previously diagnosed type 2 diabetes mellitus

## Discussion

This study shows, for the first time, a high prevalence of MS (11.8%), based on the IDF2005 definition, in Ningxia original rural population (Han ethnic group, 10.3%; Hui ethnic group, 13.7%). The results obtained in our study are similar to that published in a general China survey [5], 9.8% (95% CI 9.0-10.6) in men and17.8% (16.6-19.0) in women using ATP III Recently, Chien [13] Sibai [14]reported that the prevalence of MS in Chinese on the hospital-based was 16.2% for men and 19.0% for women; the overall prevalence of the MS in adult Lebanese population was 31.2% based on the IDF definition; In USA, the age-adjusted prevalence of MS were 23.7% from the Third National and Nutrition Examination Survey using ATP III [15]; there were 21.0% Omani adults with MS using ATP III [12]. These findings show that the MS have become serious public health challenges for Ningxia original rural population.

The prevalence of the MS was higher among Hui ethnic group compared with Han ethnic group in Ningxia original rural population. Among different Americans, the prevalence of MS in African-Americans and Mexican-Americans were higher than other ethnic groups [15-17]. The discrepancy in a variety of ethnic groups was likely due to the difference in body fat between these ethnic groups and others. In Ningxia rural area, Hui ethnic group generally have their special eating habits, overweight and obesity are more common among them. In our study, the greatest difference observed between the two ethnic groups was the prevalence of overweight (17.9% in Hui ethnic group versus 14.7% in Han ethnic group), this indicates that the link between lifestyle can be crucial factor for MS. However, the prevalence of the MS increased with the age in both ethnic groups. Other researchers have also reported similar effect of age on the prevalence of MS [4,6,12,18,19]. This increase can be attributed to a similar age-related trend in all components of MS.

Over half of rural adults have abnormality in HDL cholesterol level, and Han ethnic group was less than Hui ethnic group. This may have several causes, such as elevated triglycerides, overweight and obesity, diabetes and physical inactivity, many of which are associated with insulin resistance [20-22]. Ningxia Hui ethnic group originated from the ancient Arabs and Persians. Alcohol consumption is known to be the key factor among diet factors to increase the possibility of abnormality in HDL level [23-25]. But alcohol drinking is rare among the traditional Islamic populations (< 3% of men in Ningxia rural area drink alcohol). So the relatively high abnormality in HDL level should be more linked with other diet factors, if such strong effects exist. Interestingly, low HDL level were also observed by Abla et.al [14] among Lebanese population (51.9% in men, 47.3% in women). Based on present results, it is impossible to make conclusion whether this similarity is due to a genetic predisposition of Arabs of Middle Eastern origin or similar lifestyle factors.

Our figure is likely to be an underestimate of the true prevalence of the MS in normal Ningxia rural population because the population studied in Ningxia is more representative of rural communities where the society consists of tribal people living together with high levels of social networking. Since 1980s and soon after the Reform and Opening in china, people's living levels were developed rapidly, leading to personal income growth, better housing, and overall improvements in the population's socioeconomic conditions.

The prevalence of abnormal obesity was remarkably higher among Hui ethnic group than Han. This might be partially explained by the difference in genetic predisposition between two ethnics. However, the influence of diet habit can't be excluded. The special cultural and social restrictions in Chinese Hui ethnic group often require them to be segregated in the whole Chinese population, especially in the undeveloped original rural areas. They are not severely influenced by the Han population living around in the same area. Meantime, actually both Hui and Han population living in the same area normally took very similar physical activities. The main difference is still diet habit. For example, Hui have preferred animal fats (butter and mutton fat) and deep-fried foods for very long history. The difference in such lifestyle might be considered as the key factor leading to the difference in the prevalence of MS, these need further investigation.

It should be noted that our results are obtained by using cross-sectional data; no simple and causal relationships can be easily concluded. The sample consists of only Ningxia rural people, and might not be represented for the whole populations in Ningxia; we mainly selected adults in rural area who were farmers. However, this population represented a large inhabitant group with average living quality with stable income and living condition, lower educational background and lower health awareness in rural area in Ningxia of China.

## Conclusions

In conclusion, the age-adjusted prevalence of MS was more than 11.8% of rural adults in Ningxia of China. The present study reveals that the MS and components of MS was higher in Hui ethnic group than that in Han ethnic group; clustering of MS components and MS increase with age. This study demonstrated that epidemic of MS is not limited to the developed countries. Thus, more developing countries should expect significant burden from chronic diseases. Such findings should be taken into account when planning new or expansion of existing health service and when implementing future chronic disease prevention and control programs.

## Competing interests

The authors declare that they have no competing interests.

## Authors' contributions

ZYH designed research, wrote paper, analyzed data and performed field works. ZY performed field works, lab works, and analyzed data and wrote paper. YJJ performed field works. LXY performed lab works. XHX performed field and lab works. JJ performed field works.

All authors read and approved the final manuscript.

## Pre-publication history

The pre-publication history for this paper can be accessed here:

http://www.biomedcentral.com/1471-2458/10/140/prepub

## Supplementary Material

Additional file 1 **Results from analysis of data collected from adult: **Mean values, SD, and percentage of subjects with the components of the metabolic syndrome (IDF) and BMI (WHO) by age group and ethnic group among 928 Han ethnic group and 684 Hui ethnic group. Click here for file
