# Supplementary material for: Prevalence of the Metabolic Syndrome among rural original adults in NingXia, China
Source: BMC Public Health. 2010 Mar 17;10:140. doi: 10.1186/1471-2458-10-140 (PMC2850343; doi:10.1186/1471-2458-10-140)
Supplement: Additional file 1 — Results from analysis of data collected from adult: Mean values, SD, and percentage of subjects with the components of the metabolic syndrome (IDF) and BMI (WHO) by age group and ethnic group among 928 Han ethnic group and 684 Hui ethnic group. [file 1471-2458-10-140-S1.DOC]

**Additional file 1: Results from analysis of data collected from adult: Mean values, SD, and percentage of subjects with the components of the metabolic syndrome (IDF) and BMI (WHO) by age group and ethnic group among 928 Han ethnic group and 684 Hui ethnic group**

|  |  |  | Age group (years) | | | | | | | | | | | | | | | | | |
| --- | --- | --- | --- | --- | --- | --- | --- | --- | --- | --- | --- | --- | --- | --- | --- | --- | --- | --- | --- | --- |
|  |  |  | 25-34 | | | 35-44 | | | 45-54 | | | 55-64 | | | 65 or over | | | Total | | |
|  | | | % |  |  | % |  |  | % |  |  | % |  |  | % |  |  | % |  |  |
|  | | | Abn | Mean | SD | Abn | Mean | SD | Abn | Mean | SD | Abn | Mean | SD | Abn | Mean | SD | Abn | Mean | SD |
| Han ethnic group | | |  |  |  |  |  |  |  |  |  |  |  |  |  |  |  |  |  |  |
| Waist circumference | | | 14.4 | 74.5 | 8.8 | 15.8 | 76.2 | 8.1 | 29.9 | 80.0 | 8.0 | 42.4 | 82.9 | 8.6 | 35.3 | 84.0 | 8.8 | 25.8 | 78.6 | 8.9 |
| BMI | | | 17.7 | 22.3 | 3.3 | 21.1 | 22.9 | 2.9 | 29.0 | 23.6 | 3.0 | 34.6 | 23.7 | 3.2 | 21.5 | 22.8 | 3.6 | 25.3 | 23.1 | 3.2 |
| Triglycerides | | | 9.9 | 1.1 | 0.6 | 9.9 | 1.1 | 0.6 | 16.1 | 1.2 | 0.7 | 17.7 | 1.3 | 0.8 | 23.5 | 1.2 | 0.6 | 13.9 | 1.2 | 0.7 |
| HDL | | | 55.8 | 1.2 | 0.2 | 45.0 | 1.2 | 0.3 | 47.5 | 1.3 | 0.4 | 42.7 | 1.3 | 0.3 | 25.4 | 1.3 | 0.3 | 46.2 | 1.2 | 0.3 |
| Diastolic BP | | | 11.6 | 73.9 | 9.7 | 17.4 | 76.9 | 9.8 | 29.8 | 80.2 | 12.4 | 38.0 | 82.3 | 11.5 | 56.9 | 84.6 | 10.6 | 25.9 | 78.7 | 11.4 |
| Systolic BP | | | 5.5 | 112.6 | 12.5 | 11.6 | 116.1 | 14.5 | 26.0 | 124.5 | 21.1 | 38.0 | 131.8 | 21.9 | 72.5 | 146.7 | 22.4 | 23.0 | 122.6 | 20.3 |
| FPG | | | 37.5 | 5.4 | 0.7 | 41.6 | 5.5 | 1.3 | 50.8 | 5.7 | 1.1 | 43.7 | 5.6 | 0.9 | 41.1 | 6.1 | 2.5 | 43.6 | 5.6 | 1.2 |
| Hui ethnic group | | |  |  |  |  |  |  |  |  |  |  |  |  |  |  |  |  |  |  |
| Waist circumference | | | 14.6 | 74.6 | 8.2 | 30.5 | 78.1 | 9.8 | 35.8 | 80.6 | 8.3 | 54.2 | 85.6 | 9.3 | 59.5 | 87..7 | 8.1 | 33.6 | 79.7 | 0.8 |
| BMI | | | 17.1 | 22.5 | 2.8 | 30.9 | 23.5 | 3.3 | 38.9 | 24.3 | 3.4 | 50.9 | 24.9 | 3.5 | 27.0 | 24.2 | 3.0 | 32.7 | 23.7 | 3.3 |
| Triglycerides | | | 9.7 | 1.0 | 0.6 | 15.3 | 1.2 | 0.7 | 22.1 | 1.3 | 0.9 | 17.8 | 1.3 | 0.7 | 35.1 | 1.7 | 1.2 | 17.1 | 1.2 | 0.8 |
| HDL | | | 58.5 | 1.1 | 0.2 | 58.9 | 1.1 | 0.3 | 53.4 | 1.2 | 0.3 | 47.4 | 1.2 | 0.3 | 48.6 | 1.1 | 0.2 | 54.9 | 1.1 | 0.2 |
| Diastolic BP | | | 12.9 | 74.5 | 9.7 | 19.8 | 78.3 | 11.6 | 34.0 | 82.0 | 12.5 | 42.4 | 84.3 | 11.6 | 55.6 | 97.6 | 6.9 | 27.3 | 80.36 | 12.0 |
| Systolic BP | | | 4.9 | 115.5 | 32.8 | 10.4 | 117.5 | 16.0 | 30.9 | 124.7 | 21.0 | 34.7 | 131.2 | 23.2 | 55.6 | 143.1 | 22.2 | 20.6 | 122.5 | 24.7 |
| FPG | | | 39.0 | 5.5 | 1.4 | 45.0 | 5.5 | 0.8 | 46.6 | 5.6 | 1.0 | 48.3 | 5.7 | 1.1 | 54.0 | 5.9 | 1.5 | 45.0 | 5.6 | 1.1 |

BP: blood pressure. Units for waist circumference in cm, BMI in kg/m^2^, and triglycerides, HDL, and FPG in mmol/l. Figures for systolic or diastolic blood pressure or FPG include those on medication for theses conditions. See RESEARCH DESIGN AND METHODS FOR description of criteria of the each component of the metabolic syndrome. Abn: abnormal components of the metabolic syndrome
